# Combined Arthroscopic and Mini-Open Subpectoral Dynamic Anterior Shoulder Stabilization With Biceps Tendon for Anterior Shoulder Instability

**DOI:** 10.1016/j.eats.2025.103863

**Published:** 2025-09-23

**Authors:** Ahmad Hany Khater, Moustafa Kamal A. Mohamed, Hesham Ahmed Abdul Galil

**Affiliations:** a Department of Orthopedic Surgery, Ain Shams University, Cairo, Egypt; b Department of Orthopedic Surgery, Helwan University, Cairo, Egypt; c Department of Orthopedic Surgery, Nasser Institute for Research and Treatment, Cairo, Egypt

## Abstract

Subcritical glenoid bone loss in anterior shoulder instability represents a clinical dilemma with no consensus on the optimal management strategy. Although bony transfer procedures provide stability in cases of critical bone loss, they are associated with notable complications. Conversely, soft-tissue techniques such as Bankart repair are often insufficient alone. This technical note introduces a safe, reproducible modification of the dynamic anterior stabilization procedure using an arthroscopic onlay technique combined with subpectoral biceps tendon retrieval and anterior glenoid fixation.

Subcritical bone loss represents a gray zone in cases of anterior shoulder instability with no consensus about definitive management.^1^ Anterior shoulder instability represents a major issue with various techniques. Although bony transfer techniques are effective in cases of critical bone loss of 21% to 25% with engaging Hill-Sachs lesions^2^^,^^3^ and show low recurrence rates, they are not benign operations and have high rates of complications such as malposition, hardware issues, and neurologic insult.^4^^,^^5^ On the other hand, arthroscopic Bankart repair is successful in cases of limited bone loss,^6^ although it has a higher recurrence rate,^7^ and some authors consider even 13.5% bone loss to be critical,^6^^,^^8^ which supports the concept of adding remplissage to Bankart repair for off-track Hill-Sachs lesions.^9^

The shoulder dynamic anterior stabilization (DAS) procedure introduced by Collin and Lädermann^10^ aims to achieve arthroscopic transfer of the long head of the biceps (LHB) through a subscapularis split and LHB fixation in an inlay position in the anterior glenoid, yielding the advantage of a soft-tissue procedure and reproducing the sling effect of the conjoint tendon transfer in the Latarjet procedure. Milenin and Toussaint^11^ first described arthroscopic DAS with LHB transfer and fixation in an onlay manner on the glenoid rim, adding a labroplasty effect by creating a bumper of the biceps over the anterior glenoid, as well as adding a hammock effect and sling effect using the biceps transfer. Arthroscopic DAS can be technically demanding, so many modifications have been added to this promising technique.^12^, ^13^, ^14^, ^15^

In this technical note, we describe a safe, fast, and reproducible modification of arthroscopic onlay DAS via subpectoral biceps retrieval and shuttling through a subscapularis split in an outside-in manner, without the need for transverse humeral ligament or rotator interval release.

## Surgical Technique

Each step of the surgical technique is illustrated in Video 1. Our indications and contraindications are listed in Table 1.

**Table 1:** Indications and Contraindications of Technique

Indications
Patients with anteroinferior instability
Patients with hyperlaxity
Patients with Bankart lesion–associated SLAP tears
Patients with anterior instability and subcritical glenoid bone loss < 15%
Contraindications
Previous biceps tenotomy or tenodesis
Critical glenoid bone loss > 20%

### Patient Setup

**Fig 1:**
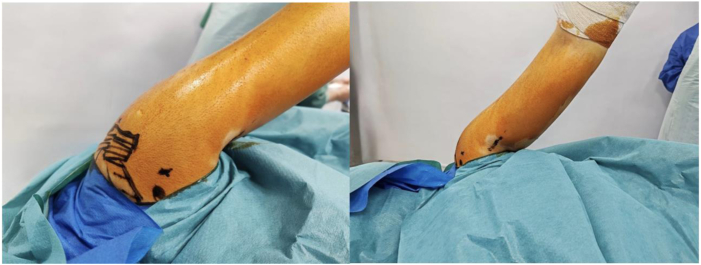
Left shoulder in lateral decubitus position showing subpectoral incision and 5-o’clock portal.

The positioning of the patient and arm is shown in Figure 1. The patient is placed in the lateral decubitus position according to surgeon preference. An examination under general anesthesia is performed to determine the direction and magnitude of instability before skin sterilization or surgical draping. The arm is positioned in traction (10-12 lb of traction for women and 12-15 lb of traction for men) at 20° of abduction and 30° of forward flexion, with a sterile roll placed in the axilla.

### Arthroscopic Approach

Four arthroscopic portals and a single mini-incision are used.

##### Posterior Portal

The posterior portal is the primary viewing portal and is created at the soft spot. Thorough diagnostic arthroscopy using a 30° scope is performed, confirming the Bankart lesion, the extent and engagement of the Hill-Sachs lesion, and rotator cuff integrity.

##### Anterior Portal

The anterior portal is the main working portal and is established from outside-in, under direct vision, using an 18-gauge spinal needle directed to the superior border of the subscapularis tendon, lateral to the conjoint tendon. A working cannula with an outflow connection (8.25-mm × 7-cm Twist-In Cannula; Arthrex, Naples, FL) is introduced through this portal. A probe is used to evaluate the Bankart lesion and biceps quality.

##### Anterolateral Portal

The anterolateral portal is used as a working and secondary viewing portal and is established from outside-in, distal to the anterolateral aspect of the acromion, using an 18-gauge spinal needle directed to the anterior glenoid margin. Another working cannula with an outflow connection (6-mm × 7-cm Twist-In Cannula) is introduced through this portal.

##### Five-O’Clock Portal

**Fig 2:**
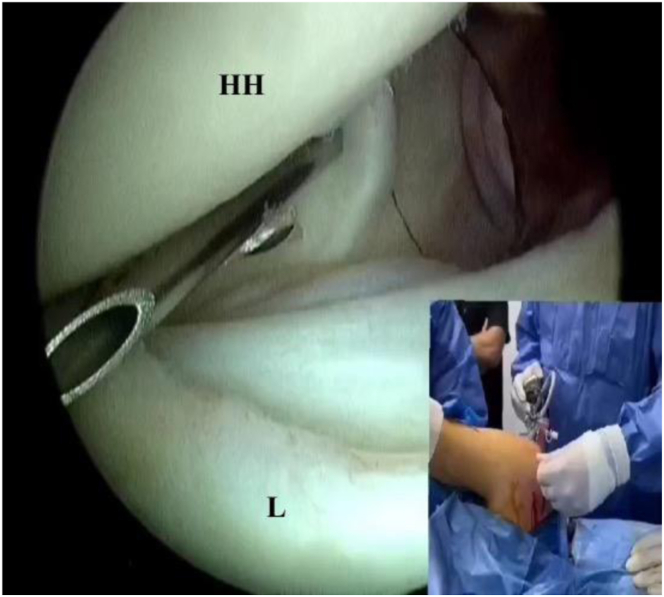
Creation of 5-o’clock portal (right shoulder): posterior portal view. A needle is introduced through the subscapularis tendon in an outside-in fashion to create the 5-o’clock portal. (HH, humeral head; L, labrum.)

The 5-o’clock portal is established from outside-in, under direct vision (Fig 1), using an 18-gauge spinal needle through the upper one-third of the subscapularis tendon. Then, a hemostat is introduced to perform a subscapularis split, which is completed from inside by radiofrequency via the anterolateral portal (Fig 2). The bony bed of the Bankart lesion is debrided using a 4 × 125-mm automated shaver (Formula Aggressive Plus Cutter; Stryker). Bankart repair is started by grasping the labrum with a suture lasso. Then, a FiberTak soft double-loaded anchor (Arthrex) is introduced from the anterior portal and inserted at the 5-o’clock position, with sequential shuttling of sutures with knot tying using a knot pusher, and the sutures are cut using a suture cutter.

The scope is introduced through the anterolateral portal, and remplissage is performed with a 5.0-mm titanium Corkscrew double-loaded suture anchor (Arthrex), but the knots are left untied until the end of surgery. The same steps for Bankart repair are repeated with a FiberTak soft double-loaded anchor inserted at the 3-o’clock position, and the knots are tied and cut.

**Fig 3:**
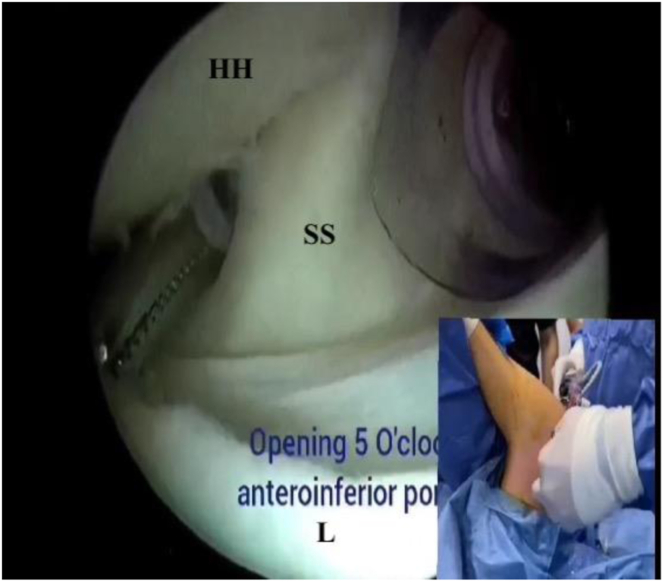
Subscapularis split with hemostat (right shoulder): posterior portal view. A hemostat is introduced through the 5-o’clock portal for the establishment of the subscapularis split. (HH, humeral head; L, labrum; SS, subscapularis tendon.)

No. 2 FiberWire suture (Arthrex) is introduced from the anterolateral portal and is shuttled with the hemostat through the subscapularis split to the 5-o’clock portal (Fig 3). Biceps tenotomy is performed with a radiofrequency device.

### Subpectoral Incision

**Fig 4:**
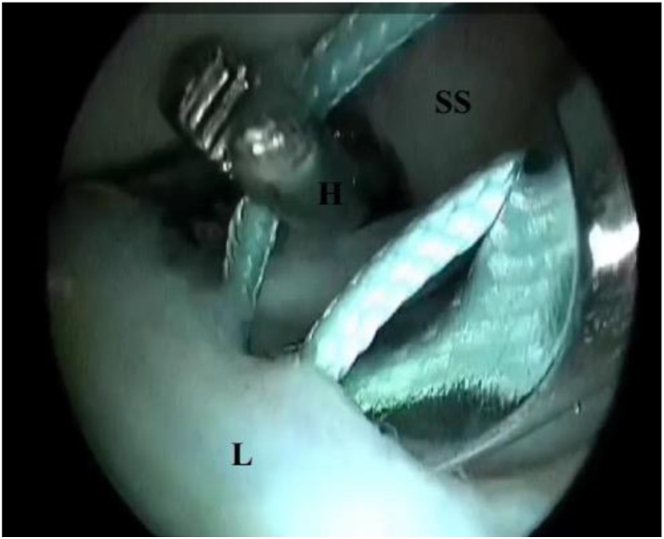
Shuttle passage through anterolateral portal and retrieval by hemostat placed through subscapularis split (right shoulder): posterior portal view. The hemostat, introduced earlier through the 5-o’clock portal, is used to retrieve a shuttle suture inserted through the anterolateral portal. (H, hemostat; L, labrum; SS, subscapularis tendon.)

**Fig 5:**
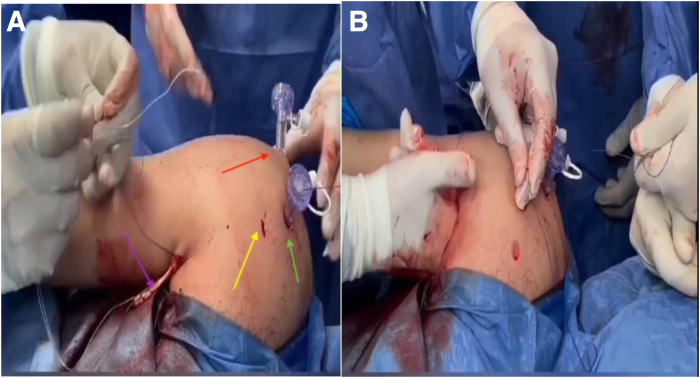
(A) Retrieval of shuttle from 5-o’clock portal anterior to subscapularis tendon (right shoulder). (B) Shuttling of long head of biceps from subpectoral incision to subscapularis split (right shoulder). The red arrow indicates the anterolateral portal; green arrow, anterior portal; yellow arrow, 5-o’clock portal; and violet arrow, long head of the biceps exteriorized through the subpectoral incision.

An incision is made at the anteromedial aspect of the humerus near the axilla, and the biceps tendon is retrieved. Whipstitching of the biceps tendon is performed using No. 2 FiberWire suture. Through the incision, the surgeon’s index finger is introduced anterior to the subscapularis to retrieve the suture passing through the split (Fig 4). This suture is used to shuttle the biceps tendon through the subscapularis split under direct visualization from the anterolateral portal (Fig 5).

### Onlay Biceps Tenodesis

**Fig 6:**
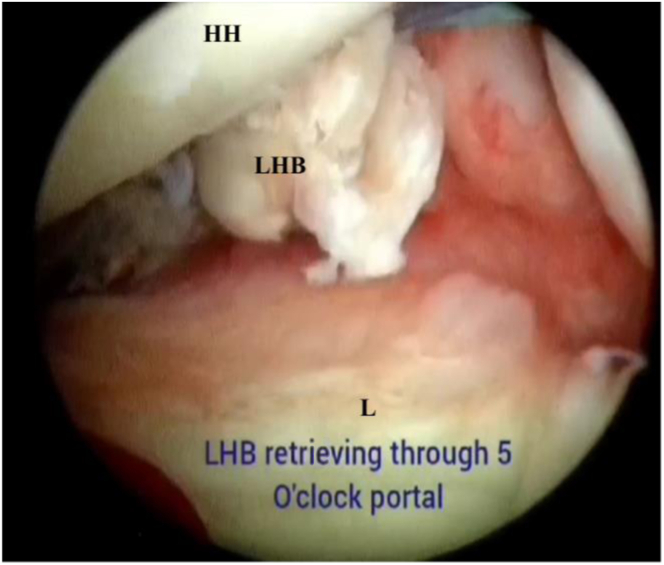
Intra-articular view of biceps retrieval through subscapularis split (right shoulder). A posterior portal view of the glenohumeral joint shows the retrieval of the biceps through the subscapularis split. (HH, humeral head; L, labrum; LHB, long head of biceps.)

**Fig 7:**
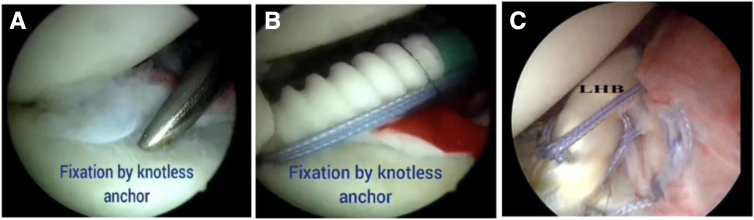
Intra-articular view of biceps onlay fixation in glenoid (right shoulder). (A) Tunnel creation for knotless anchor. (B) Biceps tenodesis with knotless anchor. (C) Final view after dynamic anterior stabilization and labral repair. (LHB, long head of biceps.)

Viewing from the anterolateral portal, a knotless anchor (SwiveLock BioComposite anchor; Arthrex) is introduced from the anterior portal at the 3-o’clock position to perform onlay biceps tenodesis, providing labroplasty and creating the sling effect, which is confirmed with direct arthroscopic visualization (Figs 6 and 7). Finally, the remplissage sutures are tied and cut under direct visualization with the arm in maximal external rotation. Advantages and disadvantages of our technique are presented in Table 2, and pearls and pitfalls are listed in Table 3.

**Table 2:** Advantages and Disadvantages

Advantages
Uses readily available biceps tendon
Double attacking SLAP lesion and anterior shoulder instability
Provides dynamic stabilization of shoulder superior to static stabilization
Quick, simple, and safer than inlay technique, which requires bone tunnels through glenoid or bulky implants (i.e., interference screws)
Subpectoral mini-open incision allows extraction of biceps tendon from groove, addressing possible concomitant long head of biceps pathology
Does not require clearance of anterior rotator interval or space anterior to subscapularis muscle, shortcutting procedure of biceps glenohumeral shuttling
Does not involve clearance of transverse ligament or rotator interval, preserving native joint anatomy
Biceps tendon allows labral augmentation
Disadvantages
Limited to patients without previous biceps tenotomy or tenodesis
Limited to patients with subcritical glenoid bone loss

**Table 3:** Pearls and Pitfalls

Pearls	Pitfalls
Perform the subscapularis split before labrum release or Bankart repair to obtain a clear view of the anterior capsule and the subscapularis.	Repairing the Bankart lesion or the capsule before creation of the subscapularis split can obscure the view.
Perform biceps tenotomy early before performing the subpectoral incision.	
Perform suture shuttling and biceps shuttling before Bankart repair.	Shuttling the suture later, after Bankart or capsular repair, could cause difficulties during suture or tendon shuttling because the biceps tendon could become entangled with the repaired tissue.
The repair shall be performed in the following order: Start with the labral preparation, followed by biceps tendon shuttling through the subscapularis split, then suture passage through the capsule and labral tissues, followed by knot tying to finish the capsulolabral repair, then finally the biceps onlay fixation. This allows for easier repair and clear visualization, as well as smooth biceps tendon shuttling and fixation.	The surgeon should avoid excessive pulling on sutures during knotless anchor introduction, which can lead to suture failure or suture-tendon cut-through, and over-tensioning of the biceps tendon, which can cause postoperative pain.
Note that tendon handling, suturing, shuttling, and delivery assisted with the mini-open subpectoral technique tend to be easier, more reproducible, and less technically demanding than other described techniques, and common suture management problems including suture entanglement are avoided.	

### Postoperative Rehabilitation

In the first 6 weeks postoperatively, a sling is worn and self-mobilization exercises are performed. Active and passive range-of-motion exercises are started at 5 weeks. Active shoulder abduction with external rotation is restricted until 6 weeks, with a return to sports recommended after 6 months. Biceps resistance training and pulling exercises are restricted until 12 weeks.

## Discussion

The short-term results of the recently introduced DAS technique show promising clinical outcomes in cases of subcritical bone loss.^16^, ^17^, ^18^ DAS offers different mechanisms to increase anterior glenoid stability: (1) the hammock effect, by pushing the lower part of the subscapularis tendon; (2) the sling effect, as the biceps force is directed posteriorly; (3) repair of the capsulolabral tissue; and (4) onlay positioning of the biceps, which produces a labroplasty effect on the anterior glenoid.

DAS is basically performed in 2 ways: inlay^16^^,^^17^ and onlay^19^^,^^20^ biceps tenodesis. The inlay method has the potential risk of jeopardizing the glenoid bone stock, whether using interference screws or metal buttons in bone tunnels. Onlay biceps tenodesis also has the advantage of labroplasty over the inlay method in cases of insufficient anterior labral tissue from recurrent instability.

Many techniques have been described for onlay DAS, but they are technically demanding and require release of the transverse humeral ligament, which may affect the subscapularis and anterior supraspinatus. Some techniques require soft-tissue clearance anterior to the subscapularis to facilitate the split and biceps shuttling, but this carries the risk of neurologic insult. Other techniques use a pulley method to shuttle the biceps, but this could lead to suture entanglement, especially for inexperienced surgeons.

In this technical note, we describe a safe, fast, and reproducible method to perform arthroscopic DAS with the onlay technique, providing a safe way to achieve a subscapularis split with no risk of neurologic insult from soft-tissue clearance anterior to the subscapularis. This technique avoids sacrificing the soft tissue in the rotator interval, which is critical for patients with instability, especially those with hyperlaxity issues. In addition, we avoid releasing the biceps from the bicipital groove, which may jeopardize the pulley and the upper subscapularis border. Our technique also offers a simple method of biceps passage through the split using the subpectoral pathway, avoiding complicated suture management in intra-articular techniques.

Our technique benefits from the onlay effect by producing a bumper that acts as a dynamic soft-tissue block on the anterior glenoid, which aids anterior shoulder stability. Moreover, the arthroscopic approach allows us to perform Bankart repair and remplissage, which enhances stability. The drawbacks of our technique are its limitation to cases with subcritical bone loss and its requirement for good-quality biceps tendon.

## Disclosures

All authors (H.A.A.G., M.K.A.M., A.H.K.) declare that they have no known competing financial interests or personal relationships that could have appeared to influence the work reported in this paper.

## Supplementary Data

Video 1 Arthroscopic subpectoral-assisted dynamic anterior stabilization (DAS) of the shoulder in a patient with recurrent anterior instability and subcritical glenoid bone loss. Diagnostic arthroscopy in the lateral decubitus position is shown, in addition to subscapularis split, shuttle suture passage, subpectoral biceps retrieval, biceps shuttling, glenoid preparation, and biceps tenodesis with an onlay fixation technique using a knotless anchor. Key steps including subscapularis split creation, biceps rerouting, and suture anchor placement are shown with clear intra-articular and subpectoral views (left side).
